# Detailed Anatomical Volumetric Study of Deep Nuclei of Brain and Other Structures Between Parkinson’s Disease Patients Who Had Deep Brain Stimulation and Control Group

**DOI:** 10.21315/mjms2020.27.3.6

**Published:** 2020-06-30

**Authors:** Chin Hwee Goh, Johari Yap Abdullah, Zamzuri Idris, Abdul Rahman Izaini Ghani, Jafri Malin Abdullah, Albert Sii Hieng Wong, John Tharakan, Salmah Mar Win

**Affiliations:** 1 Department of Neurosciences, School of Medical Sciences, Universiti Sains Malaysia, Kubang Kerian, Kelantan, Malaysia; 2 Brain and Behaviour Cluster, School of Medical Sciences, Universiti Sains Malaysia, Kubang Kerian, Kelantan, Malaysia; 3 Biom3D Laboratory, School of Dental Sciences, Universiti Sains Malaysia, Kubang Kerian, Kelantan, Malaysia; 4 Department of Radiology, School of Medical Sciences, Universiti Sains Malaysia, Kubang Kerian, Kelantan, Malaysia; 5 Department of Neurosurgery, Sarawak General Hospital, Kuching, Sarawak, Malaysia

**Keywords:** brain volumetry, brain morphometry, Parkinson’s disease, basal ganglia, globus pallidus, subthalamic nucleus

## Abstract

**Background:**

Deep brain stimulation (DBS) was pioneered by Neuroscience team of Hospital Universiti Sains Malaysia (HUSM) nearly a decade ago to treat advanced medically refractory idiopathic Parkinson’s disease (IPD) patients.

**Objectives:**

Brain volume reduction occurs with age, especially in Parkinson plus syndrome or psychiatric disorders. We searched to define the degree of volume discrepancy in advanced IPD patients and correlate the anatomical volumetric changes to motor symptoms and cognitive function.

**Methods:**

We determined the magnetic resonance imaging (MRI)-based volumetry of deep brain nuclei and brain structures of DBS-IPD group and matched controls.

**Results:**

DBS-IPD group had significant deep nuclei atrophy and volume discrepancy, yet none had cognitive or psychobehavioural disturbances. Globus pallidus volume showed positive correlation to higher mental function.

**Conclusion:**

The morphometric changes and clinical severity discrepancy in IPD may imply a more complex degenerative mechanism involving multiple neural pathways. Such alteration could be early changes before clinical manifestation.

## Introduction

Parkinson’s disease (PD) is a chronic progressive neurodegenerative disorder characterised by cardinal clinical symptoms of bradykinesia, resting tremor, rigidity and postural instability. It is first described by James Parkinson in his classical 1817 monograph ‘An essay on the shaking palsy’ (1).

This disease affects around 100 per 100,000 population over the age of 40 and remains a clinical diagnosis as there are still no laboratory tests to date to confirm the diagnosis. Neuroimaging is usually unrevealing and often performed to exclude mimics of PD or secondary Parkinsonian syndromes.

Hospital Universiti Sains Malaysia (HUSM) pioneered the first deep brain stimulation since two decades ago. It has since then provided surgical treatment to medically refractory advanced idiopathic Parkinson’s disease (IPD) and other movement disorders. Although deep brain stimulation (DBS) is still a surgical procedure dictated by resource availability in the University Hospital, it has created an advantageous though limited opportunity to conduct volumetric brain evaluations on Parkinson’s disease patients who had DBS. It is known that atrophy or volume reduction occurs with age, we search to define the degree of volume discrepancy in PD patients and to correlate the anatomical volumetric changes to the clinical severity of the disease.

## Methods

**Figure 1:**
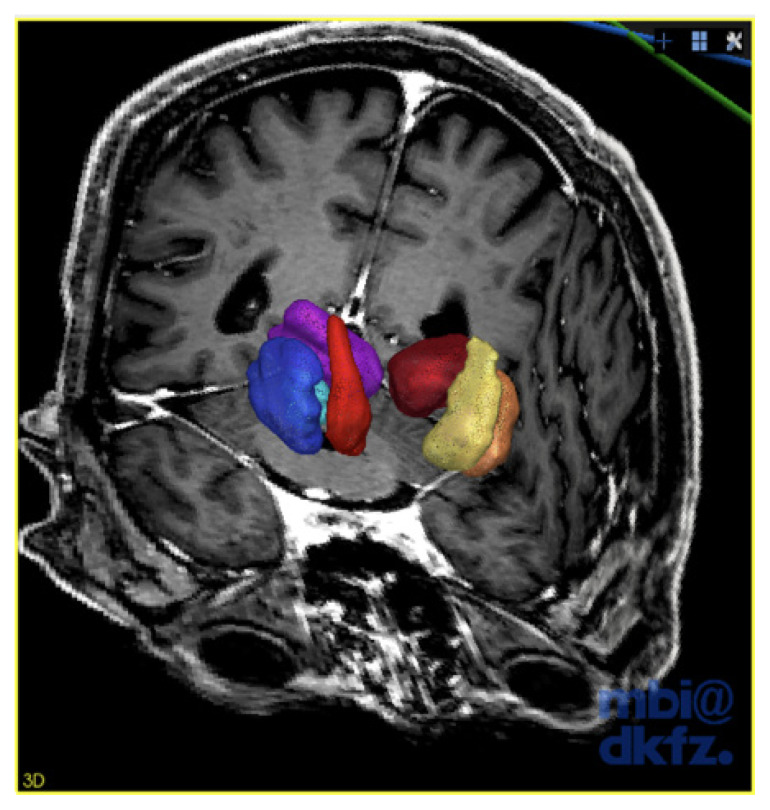
The deep nuclei of the brain 3D reconstruction model

**Figure 2:**
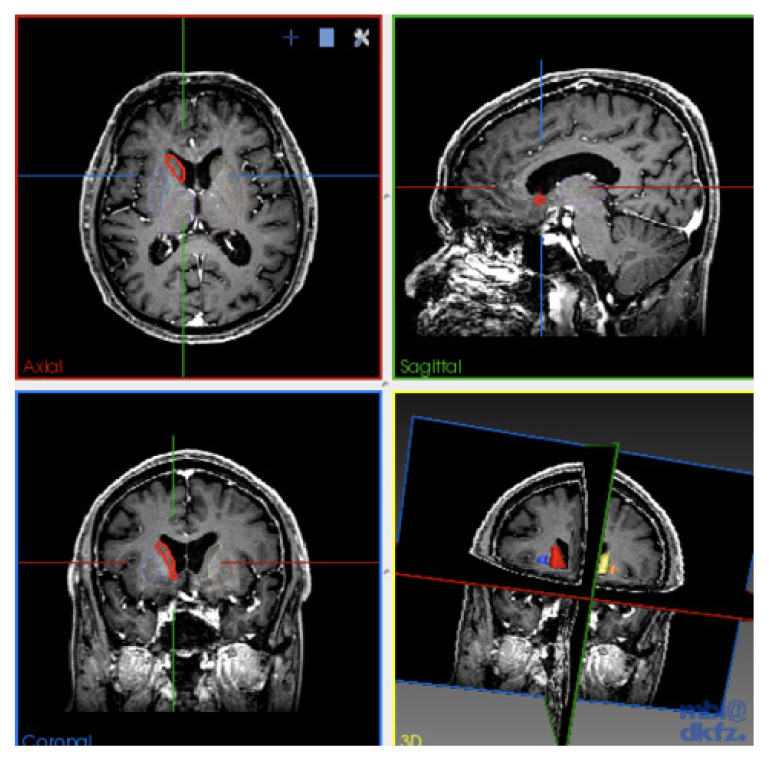
Multiplanar view for delineation of the ROI and summated to 3D model

This was a retrospective observational magnetic resonance imaging (MRI) brain volumetric study of nine medically refractory advanced IPD patients. They had undergone deep brain stimulation (DBS-IPD) in HUSM. The disease was defined by Hoehn and Yahr scale. Associated Parkinson plus, dementia and psychiatric disorders were excluded. Persons who were neurologically intact and had MRI brain done were selected from the hospital patient database as controls. They were matched to the cohort according to age. The studied nuclei and brain structures were globus pallidus (GP), subthalamic nucleus (STN), head of caudate nucleus (HCn), putamen (Pn), thalamus (Th), head of hippocampus (Hh) and amygdala (Amd). We utilised a validated open-source software (C++)—the Medical Imaging Interaction Toolkit (MITK) for volumetry analysis. This was done based on 3.0 Tesla MRI (Philips, USA) T1-weighted three-dimensional (3D)-fast spoiled gradient-recalled multi-planar (TR 7.6ms, TE 3.4ms, slice thickness 1 mm, no gap, matrix 220 218, FOV 240 mm), and T2-weighted spin-echo axial and coronal (TR 3000 ms, TE 80 ms, slice thickness 1 mm, no gap, matrix 400 255, FOV 230 mm). These regions of interest (ROI) were defined and measured in Digital Imaging Communications in Medicine (DICOM) format. The ROIs were marked by using a combination of automated segmentation tool and manually traced employing computer mouse-driven pointer. Manual trace was based on the exact delineation of structures in axial plane supplemented by multi-planar coronal and sagittal views. Parameters such as size, contrast and brightness were adjusted to achieve sharp border of the ROI. The images were interpolated in three dimensions (Figures 1 and 2). The summation was processed in exact cubic millimetre (cm^3^). The Schaltenbrand and Wahren’s Atlas of stereotaxy of human brain, and 7.0 Tesla MRI brain atlas in vivo with cryomacrotome correlation were used as an adjunct to enhance the accuracy of ROI hinged on contrasts between white and grey matter with correct sequence T1W gadolinium delineating the deep nuclei contours. Descriptive statistics such as mean with standard deviation and median were used to illustrate the characteristics of the cohort. Univariate analysis Mann-Whitney U test was used to determine the non-parametric variables. *P* ≤ 0.05 was considered statistically significant. Spearman rank order correlation coefficient would be applied for bivariate analysis. All data were analysed by using SPSS version 22.

## Results

### Demographic

The DBS-IPD group age ranged 52 to 70 years old. They were multiracial distribution and predominantly Malay. Six were males and three females. Mean duration from symptoms to diagnosis was three years though the majority diagnosed less than a year. The mean age diagnosis was 47.3 years. All the subjects progressed to Hoehn and Yahr stage 4 disease over 6 to 25 years, and mean duration was 13.9 years. They did not have Parkinson plus features. Mean score of mini-mental state examination (MMSE) was 26. All the DBS-IPD cases were on a concoction of Parkinson’s disease medications. Demographic data of surgical group were summarised (Table 1).

### Data Characteristics

Visual inspection of histograms and normal Q-Q plots showed the cohort were not normally distributed with slight skewness and kurtosis.

### Mean and Median Volume

The DBS-IPD group had an overall smaller deep nuclei of brain structures compared to control. This included HCn (DBS-IPD = 1605.5 mm^3^, Control = 2214.0 mm^3^), GP (DBS-IPD = 935.0 mm^3^, Control = 1180.0 mm^3^), Pn (DBS-IPD = 3167.5 mm^3^, Control = 3472.0 mm^3^), Hh (DBS-IPD = 924.5 mm^3^, Control = 1186.0 mm^3^) and STN (DBS-IPD = 103.0 mm^3^, Control = 139.0 mm^3^). A Mann-Whitney U test indicated that the differences were statistically significant, HCn (U = 47.00, z = −3.64, *P* < 0.05), GP (U = 58.00, z = −3.29, *P* < 0.05), Pn (U = 92.50, z = −2.10, *P* < 0.05), Hh (U = 33.00, z = −4.08, *P* < 0.05) and STN (U = 0.01, z = −5.128, *P* < 0.05). STN, GP, HCn and Hh demonstrated large effect size by Cohen’s classification (more or equal to 0.5). However, Th and Amd volumes were comparable in DBS-IPD and control groups. The distributions in the two groups were not differed significantly, Th (U = 160.00, z = −0.06, *P* = 0.950) and Amd (U = 152.00, z = −0.32, *P* = 0.752) (Table 2).

### Volume Discrepancy

Subgroup analysis of the structure by laterality showed that the GP and STN were substantially reduced with volume discrepancy, 23% and 29% (left and right) in the former, 33% (both) in the latter. The discrepancy was statistically significant, both GP [U(N_DBS-IPD_=9, N_Control_=9) = 14.00, z = −2.34, *P* < 0.05] and both STN [U(N_DBS-IPD_=9, N_Control_=9) = 0.01, z = −3.58, *P* < 0.05]. HCn had the most volume discrepancy measured at 35% and 31% for left and right. The differences were significant for left HCn [U(N_DBS-IPD_=9, N_Control_=9) = 10.00, z = −2.70, *P* < 0.05] and right HCn [U(N_DBS-IPD_=9, N_Control_=9) = 15.00, z = −2.25, *P* < 0.05]. Hh measured as the proximal third of the entire length was also smaller compared to control group. Its volume reduction discrepancy was 26% (left) and 32% (right). This differed statistically significant for left Hh [U(N_DBS-IPD_=9, N_Control_=9) = 6.00, z = −3.05, *P* < 0.05] and right Hh [U(N_DBS-IPD_=9, N_Control_=9) = 10.00, z = −2.70, *P* < 0.05]. Pn showed the least volume discrepancy. Its volume reduction discrepancy was measured at 16% and 5% (left and right). Mann-Whitney U test only showed statistically significant difference in left Pn, [U(N_DBS-IPD_=9, N_Control_=9) = 15.00, z = −2.21, *P* < 0.05]. There were no significant discrepancies among Th and Amd (Table 3).

### Higher Mental Function, UPDRS Motor Score III, Age and Laterality (Left and Right)

Spearman rank order correlation coefficient revealed a moderate, positive correlation between GP and higher mental function (MMSE). Higher scores of MMSE were correlated with a more prominent volume of GP, *r* = 0.494, *n* = 18, *P* = 0.037. However, this was not affected by the volume discrepancy. There was collinearity observed between deep nuclei and post-operative unified Parkinson’s disease rating scale (UPDRS) motor score III with low correlation, namely head of caudate nuclei (*r* = 0.240, *P* = 0.337), putamen (*r* = 0.321, *P* = 0.194), globus pallidus (*r* = −0.0232, *P* = 0.354) and amygdala (*r* = 0.232, *P* = 0.354), though statistically not significant. The *P*-values were likely due to small sample size. Nonetheless, there was no correlation between volume discrepancy and age. This finding was similar in the laterality of studied nuclei and brain structures.

## Discussion

Traditionally, studies of brain morphology entirely relied on autopsy material. In recent advances of clinical neuroscience, brain morphometry has emerged as one of the most dynamic fields with the development of scientific computation simulation, technologies and advanced MRI resolution. The era of MRI-based morphometry expansion has become a valuable tool for studying human brain plasticity in vivo. This immense advantage enables the invivo observation of brain morphology and the correlation with brain function physiologically (2).

Voxel-based morphometric studies have shown brain atrophy to exist in many cortical and subcortical regions, particularly in the basal ganglia (caudate nucleus, putamen, globus pallidus, subthalamic nucleus, substantial nigra) (3). Some studies also revealed certain brain areas (frontal lobe, temporoparietal junction, parietal lobe, insula, anterior cingulate cortex and thalamus) had compensatory increase volume in PD patients (4–5). However, the results on cerebral region atrophy were somewhat inconsistent, and such variability among the findings remained uncertain. Although PD has primarily been viewed as associated with functional changes, studies had suggested that the accurate interoperation of the significance of such functional imaging studies depends on determining whether volume changes occurred in various deep nuclei of the brain (6). The literature widely reported brain structures morphologic changes in early stages of PD. These volume reductions varied according to region of brain and were associated with Parkinson-plus syndrome, dementia and psychiatric disorders (7–9).

On the contrary, our series of DBS-IPD patients were advanced late-stage PD. They did not have psycho-behavioural changes or Parkinson plus syndrome despite showing a significant mean volume reduction discrepancy of 25%, especially HCn and GP. Striatum is known to involve in cognitive functions, and globus pallidus plays a role in attention and action inhibition (10). Functional MRI revealed evidence of globus pallidus distinct roles in planning and movement parameters of precision grip. Moreover, all of our DBS-IPD patients had no impaired higher mental function. This implied that PD might not be a disease that was solely restricted to the nigrostriatal pathway but brain structures beyond that, even at early stages. Gillies et al. (11) utilised natural experiment offered by DBS to compare GP interna local field potential responses in PD. The electrophysiology study suggested there was more than one mechanism to basal ganglia-dependent cognitive function, allowing Parkinsonian basal ganglia to still act as a moderator to thalamocortical circuits despite the striatonigral pathway degeneration. Another study reported that pathological non-dopaminergic neuronal system changes had caused multiple neuromediator and neurochemistry dysfunctions that accounted for the intricate patterns of functional deficits (12). This included cholinergic, serotonergic, peptinergic and noradrenergic systems.

The discrimination of morphologic changes in deep nuclei might represent the early signs of disease progression ahead of neurochemistry and clinical manifestations. The analysis of diffusion tensor imaging further explained the presence of widespread micro-structural damage seen in early stage of PD before progression of the cognitive dysfunction (13). Few morphologic research studies revealed that PD without dementia also demonstrated grey matter volume changes in early stage of disease. Krabbe et al. (14) reported 10%–15% volume reduction of caudate nucleus, putamen, hippocampus and amygdala in early-stage PD without dementia.

Interestingly, we did not observe volume changes in thalamus and amygdala. These two structures play a crucial role in cognition and affection, respectively. The volumes of the two structures were comparable in both groups. Various literatures supported this finding (15). This suggested that the two had better durability and resistance to neurodegenerative effect or simply not yet achieved the noticeable changes in volume even in advanced stage of PD.

## Conclusion

Significant deep brain nuclei volume reduction was seen in advanced PD without cognitive or psycho-behavioural disturbances. This morphometric changes and clinical severity discrepancy in PD may indicate a more complex degenerative mechanism involving multiple neural pathways. Current study had demonstrated such anatomical alteration preceded clinical neurophysiological manifestations, although the degree of volume discrepancy did not affect its clinical severity.

## Figures and Tables

**Table 1:** Demographic of the DBS IPD group

Surgical group	001	002	003	004	005	006	007	008	009
Age	52	53	53	53	54	61	67	67	70
Sex	Male	Male	Female	Female	Male	Male	Male	Female	Male
Race	Malay	Malay	Chinese	Malay	Indian	Chinese	Malay	Malay	Malay
Occupation	School headmaster	Telekom executive	Housewife	Government officer	Businessman	Businessman	Teacher	Religious guru	Teacher
Parkinson’s disease (Hoehn and Yahr)									
Symptom onset (year)	1990	1990	1998	1998	1995	1997	2003	2002	2004
Date of diagnosis	1997	2000	2001	1999	1996	1997	2003	2004	2007
Duration of progress (years)	25	25	7	10	13	17	6	11	11
Medication (Pre-DBS)	Madopar, Amdantadine, Pramipexole, Entacapone	Madopar, Comtan, Trivastal	Stalevo, Amantaadine, Trivastal, Comtan	Madopar, Ropinirole, Amantadine, Piribedil	Madopar, Artane, Quetipine, Ropinirole	Stalevo, Rotaglotine, Amantadine	Stalevo, Ropinirole	Sinemet, Amantadine, Artane	Stalevo, Requiep
Education level	Tertiary	Tertiary	Secondary	Secondary	Tertiary	Secondary	Tertiary	Tertiary	Tertiary
Handedness	Right	Right	Right	Right	Right	Right	Right	Left	Right
DBS surgery	2015, Bilateral STN	2007, Bilateral STN	2010, Bilateral STN	2010, Bilateral STN	2010 Bilateral STN	2013, Bilateral STN	2013, Bilateral STN	2016, Bilateral STN	2015, Bilateral STN

**Table 2:** The median and mean volume of deep nuclei and other brain structures between DBS-IPD and control group

Region of interest	Surgical group (DBS-IPD) Volume, *n* = 18		Control group Volume, *n* = 18		U	Z	*P*-value
	Mean (SD), Median	Mean rank	Mean (SD), Median	Mean rank			
Caudate head of nucleus	1625.5 (419.25), 1605.5	12.11	2159.2 (290.07), 2214.0	24.89	47.00	−3.639	0.000
Putamen	3145.2 (477.67), 3167.5	14.64	3467.8 (311.64), 3472.0	22.36	92.50	−2.100	0.028
Globus pallidus	957.3 (114.90), 935.0	12.72	1207.9 (259.21), 1180.0	24.28	58.00	−3.290	0.001
Subthalamic nucleus	103.6 (7.75), 103.0	9.50	137.7 (6.83), 139.0	27.50	0.01	−5.128	0.001
Thalamus	5117.4 (530.78), 5042.5	18.39	5055.2 (780.54), 5281.5	18.61	160.00	−0.063	0.950
Hippocampus	931.9 (138.56), 924.5	11.33	1200.1 (162.92), 1186.0	25.67	33.00	−4.081	0.000
Amygdala	1885.6 (295.65), 1928.5	19.06	1849.94 (265.3), 1842.5	17.94	152.00	−0.316	0.752

Note: The highlighted *P*-values indicated significant result (< 0.05)

**Table 3:** Volume discrepancy of deep nuclei

	Left				Right			
Region of interest	Volume discrepancy (%)	Mann- Whitney U	Z	*P*-value	Volume discrepancy (%)	Mann-Whitney U	Z	*P*-value
Caudate head of nucleus	35.1	10.00	−2.695	0.007	30.6	15.00	−2.252	0.024
Putamen	15.8	15.50	−2.209	0.027	5.1	33.00	−0.662	0.508
Globus pallidus	23.1	14.00	−2.300	0.019	29.4	14.00	−2.300	0.019
Subthalamic nucleus	32.6	0.001	−3.578	0.001	32.6	0.001	−3.580	0.001
Thalamus	0.01	36.00	−0.397	0.691	2.4	40.00	−0.044	0.965
Hippocampus	32.3	6.00	−3.046	0.002	25.5	10.00	−2.693	0.002
Amygdala	3.5	35.00	−0.486	0.627	0.3	40.50	0.000	1.000

Note: The highlighted *P*-values indicated significant result (< 0.05)
